# President’s Address before the Southern Surgical and Gynecological Society, Atlanta, November 11, 1890

**Published:** 1891-01

**Authors:** Geo. J. Englemann

**Affiliations:** President Southern Surgical and Gynecological Association; President St. Louis Obstetrical and Gynecological Society; Professor Diseases of Women, Missouri Medical College and Post-Graduate School; Fellow of American Gynecological Society; Fellow British Gynecological Society; London Obstetrical Society, etc.


VOL. VII. JANUARY, 1891. NO. 11.

### Original Communications.

### PRESIDENT'S ADDRESS BEFORE THE SOUTHERN SURGICAL AND GYNECOLOGICAL SOCIETY, ATLANTA, NOVEMBER 11th, 1890.

### CAUSES THAT IMPERIL THE HEALTH OF THE AMERICAN GIRL AND THE NECESSITY OF FEMALE HYGIENE.

By GEO. J. ENGLEMANN, A. M., M. D.,

President Southern Surgical and Gynecological Association; President St. Louis Obstetrical and Gynecological Society; Professor Diseases of Women, Missouri Medical College and Post-Graduate School; Fellow of American Gynecological Society; Fellow British Gynecological Society; London Obstetrical Society, etc.

To guide woman in greater safety through the dangers which beset her path in life is one of the highest and most sacred duties of our profession; as we may say that the care of woman is the care of the nation; the good health, mental, moral and physical, of the woman and mother is the very foundation of our national growth and prosperity, and the conditions which tend to undermine that health cover a field of such extent and importance that you will readily realize the necessity of a limitation of my remarks.

### GIRL.

I shall speak to you of the girl, the coming mother, of adolescence, the most important and interesting period of woman's life; the period of greatest functional activity, during which the foundation for future health is laid. It is the most dangerous period, during which the organism, the budding mind, the developing system, are more susceptible to disturbing influences from without and within.

It is the time when the clay is soft and the vessel is forming, when it yields most readily, and trifling impressions are permanently recorded.

It is in this period of school, the period of beginning social life, the period of learning in trades, that the nervous energies of the female are most fully engaged, and her activity is concentrated on the brain function, to the detriment of other functions; above all, the developing sexual function, the central and most important, and at that time most readily disturbed.

That I speak of the American girl is but natural, and I need hardly say that she is, moreover, subjected to a far greater number of disturbing influences than her sister in other lands, more recklessly exposed to the very injuries which react most violently upon the female function.

True as it generally is that woman is the exponent of a nation, indicative of its development, of its growth or depreciation, the American woman is more closely linked with the state and fate of her nation than the women of other countries.

She shares the febrile activity of our existence ; she is the factor in our social and political economy; she participates in the rush and crush of the times to the utmost extent of her nerve force and brain power ; but especially is this true of the American girl, as compared with the girls of other countries.

### AMERICAN GIRL.

When I speak of the American girl, I speak not of the extremes, not of the rich or the poor, but of the girl of the great middle class of our cities—the typical American girl. Compare her to her hearty, strong-boned English sister; the French girl, raised within convent walls, carefully guarded, removed from life until her marriage; or to the average German girl, reared amid the calmness of her surroundings, taught the solid rudiments of learning, and educated in household duties.

Compare her even to her sister of the village or country, if she be still free from contaminating influences—from the nerve life of city or boarding school.

You will recognize her at once. You will recognize the effect of brain work and nerve strain, the rush and mental anxiety of the day, the want of muscular training, the want of harmony in life, in training and education, mental, moral and physical.

Whilst I will not agree with Ploss in his characteristics of the American girl, when he records the statement of an ungallant Yankee, that the American girl has no bones, no muscle, no vitality—only nerves; and "what should we expect," he asked, "when in the place of bread they eat chalk, in the place of wine they drink ice-water, wear tight corsets and shoes?"

Even now, one of our greatest authorities and one of our keenest observers, Dr. T. Weir Mitchell, says that the American woman is unfit for her duties as a woman, not quite up to what nature asks of her as a wife and mother.

I believe that the essential causative factor to which the ill-health of the American girl must be referred is functional neglect, or ignoring of her functions, and over brain work, over exertions of the nerves and emotions, with imperfect development of the muscular system, an inharmonious development and exertion, physical, mental and moral.

The peculiar organization of woman is too much ignored, and it is claimed that woman is equal to man in her primitive state, and that her functions are physiologically and naturally not in want of any particular attention. Is this function of such paramount importance, and does it so completely control woman's life? This is a physiological problem upon the solution of which depends the relative capacity of woman for labor, mental and physical, and an understanding of the causes of diseases, their influence and their remedies. The answer is readily found, if we observe with an unprejudiced eye the existing conditions.

### IMPORTANCE OF THE SEXUAL FUNCTION.

Throughout all the great kingdoms of nature, the importance of the reproductive function in the female is demonstrated ; it is strikingly demonstrated in the vegetable as well as in the animal life; it was recognized by the intuitive keenness of the most primitive people, and distinct expression is given to these fundamental facts in nature by the great law-givers of ancient times.

### PLANTS.

Differences in sex are more or less well marked throughout the vegetable kingdom, and the supremacy of the reproductive function in the female, with the necessity of additional vitality for its perfect performance, is distinctly characterized.

This is well exemplified in our common hemp, which develops over fifty per cent. of male plants, when the seed is fairly distributed over fertile soil, as a superabundance must be provided for the necessary waste which follows distribution of the male pollen by the winds. If the seed are thickly sown, so that the nutrition is insufficient or scant, the number of female plants will be diminished, as the supply requisite for their greater vitality is wanting; and if densely crowded, the female plant may be altogether unable to develop. To the fruit grower the great demand of vitality for the reproductive function is well known. The apple tree with a luscious growth and foliage bears no fruit, its vitality all being directed towards the one function of vegetable growth; to reduce this, the tree is girdled, when with a diminution of growth and foliage it again fruits.

So, harmonious development of the function is as necessary for the symmetrical growth of the plant, as it is to the perfect development of the human being.

### CUSTOMS OF RACES.—ANCIENTS.

The high importance of the peculiar function of women, which it is a tendency of our enlightened nineteenth century to undervalue and ignore, was fully appreciated by the people of olden times, and the necessity of functional hygiene for the welfare of the community was recognized to such an extent that it was made obligatory by laws of custom or religion ; and the highest penalties—expulsion from the community, everlasting damnation and even death—were imposed for certain transgressions which are thoughtlessly practiced to-day by the refined and enlightened beings of our advanced civilization.

The essence of such laws and customs of the savages of to-day, in fact of all primitive people from past to present, was rest, functional rest.

Instinct and experience have taught primitive people these truths, which in our day are but imperfectly realized, even by medical science, and denied by some who call the susceptibility of the woman of to-day and her ailments unnatural, and claim them to be altogether the result of civilization.

They claim that woman in her natural state is the physical equal of man, and constantly point to the primitive woman, female of savage people, as an example of this supposed axiom.

Do they know how well the same savage is aware of the weakness of woman and her susceptibilities at certain periods of her life, and with what care he protects her from harm at these periods, so that health may be retained? The aid of religious superstition and the anger of the gods was invoked to secure this simple, but effective female hygiene, to secure the much needed rest. Rest by isolation during the periods of functional activity, up to nine days each month, up to thirty and ninety after childbirth, and up to five months at puberty.

PUBERTY.

The budding of the maid into womanhood is marked by a long period of rest and isolation, and her return to her tribe is celebrated by ceremonies of various kinds.

The importance of surrounding woman with certain precautions during the height of these great functional waves of her existence was appreciated by all people living in an approximately natural state, by all races at all times, and among their comparatively few religious customs this one, affording rest to women, was one of those most persistently adhered to.

This idea has been so deeply impressed that a mere touch is looked upon as contamination, and she is accordingly obliged to desist from all the ordinary duties of life, and is removed from its exertions and excitements by forced isolation. Where isolation is not customary, as we find it among people approaching civilization, a certain characteristic mark or signal is worn, for the wearer a passport of safety; in East India young girls show their condition by a small piece of linen steeped in blood, which is worn at the neck, as I have myself seen in the Nautch girls brought to this country for the purpose of exhibition; and the Woloff negress wears a bright colored folded cloth upon the cheek.

In the same manner the necessary rest is accorded woman for the period of susceptibility from three to four and even five more days each month throughout functional life.

We either find that a hut is erected at some distance from the village as among the Bedas, in Cambodia and on the Isle of Yap, one of the west Caroline Islands, or that a certain house is assigned for the purpose, as a place of seclusion within the village; so in New Caledonia, upon the coast of Guinea, among the Caffres, the Hottentots and the American Indians; the Hindoos, the Nayers of Malabar and others, assign to the woman, in families favorably situated, a separate room in the house.

In Japan likewise, she is confined in a separate room, not permitted to eat with the family, and forbidden even the visiting of Temple, admitting no possible excuse for leaving the house.

Work of every kind, and the bath is strictly forbidden, the dangers of cold water at this time being thoroughly appreciated by all these people, whilst it is a necessary part of their religious teachings, that before returning to the village and their families, after the wave has passed, a bath is taken. The laws of Moses and Zoroaster are almost identical, pointing to these great functional waves of woman's life as a working of the gods.

The laws of Zoroaster necessitated a seclusion of four nights for woman, and, what is remarkable, she was then forced to determine her condition by examination, and if the flow had not completely ceased, indicating an abnormity, additional precautions were observed; she must remain five more nights to which nine days were added, after which time she might cleanse herself and return to life.

### IMPORT OF FUNCTIONAL MENSES.

The life of woman does not run smoothly like that of man. It is characterized by marked periodicity, by ebbs and floods, by great life waves, which are dominant in the sphere of her especial functions; waves of vascular tension and nerve excitement, marked by a heightened activity and susceptibility of her entire being, distinctly indicating that woman's periodical activity is not a local process as we have been taught, but one involving the entire female organization, as it was held to be by the ancients, and exerting a permanent influence upon that organism of whose condition and development it is indicative. This function of woman involves the entire vascular and nervous system, and may be said to be the central exchange of that great network of wires, the vaso-motor-nerves, the great sympathetic, linking it most intimately with the brain and spinal cord, so with every part of the system.

### WAVE.

The most persistent period of nerve and vascular excitement is that of developing womanhood, when for months the system is in a period of unusual activity, and consequently the highest susceptibility, which does not cease as speedily as we see its outward tokens disappear. Then follow the cyclical changes of mature activity, varied by higher waves of active reproduction.

The functional wave slowly rises until it reaches flood height, with an increase of nerve activity and vascular tension, accompanied by a rise of temperature, as Mary Putman Jacobi tells us, of from 0.01 degrees to 0.8 degrees F. and it is during the decline of this wave that the depletion takes place, when the distended vessels rupture and nature relieves herself, the temperature steadily sinking, but not reaching the normal until after the cessation of all external symptoms.

Are we to believe that a function which so deeply implicates the entire system can be so completely disregarded ? That it does not demand special care—greater care than functions less general, less susceptible, less intimately connected with the organism ?

Are we to believe that this function can be ignored; are we to be guided by the dangerous arguments of those who claim that precautions are unnecessary at this period ?

IS WOMAN EQUAL TO MAN.

In considering the initial causes of disease in their effect upon woman, we must analyze not alone this one function, but we must consider the entire being of woman, that we may apply the proper standard, and that we may not measure her capacity for labor, mental or physical, or her powers of endurance, with those of man. Woman, the woman of our civilization, cannot be properly compared with man; she is differently organized, differences of many kinds exist—the most obvious, of course, external and anatomical. Form and shape differ; her organism is a different one; individual organs are said to be more vascular and more nervy; she is more emotional, more readily exhausted; less able to bear continuous and prolonged application; more blood is produced, the circulation is more active, so also the respiration. The period of puberty is shorter and more marked, and the last stages of development are reached at an earlier period in life.

Consider her lighter frame, her nervous organization, her emotional nature ; consider the constant activity of the reproductive functions, the influence of this sphere which dominates her entire being ; the intimate connection of every organ, above all the spinal cord, with this reproductive center; and then, need we wonder that injury befalls this sensitive organization when exposed to the intense and continuous nerve and muscle strain of our present system of education and labor, upon a basis of male vigor?

STATE OF HEALTH.

Whilst the evil is great, I am happy to say that an improvement in the physique of the American girl of the better classes has become distinctly evident of late years, due to progress of the science of hygiene, and its better understanding by the educated public, and perhaps to the introduction of sound physiological and hygienic doctrines in some of the more advanced schools, but above all to the new fad, to the increased popularity of outdoor sports.

In the higher classes we mark this change, and we may thank a benign Providence for the change of fashion which has produced this result. The girl must have a good color, a healthy figure, a brisk walk, to be in the swim; riding and walking, lawn tennis and rowing, even fencing, have become fashionable, and are working wonders upon the health of the American girl who can afford these luxuries—the same girl who twenty years ago drank vinegar to acquire a fashionable pallor, and an early grave.

Compare the swinging gate of the girl of to-day with the mincing walk and the Grecian Bend of some years ago.

A beginning has been made, but the greatest difficulties are still to be overcome; the American girl has a just claim to the most perfect and harmonious development, mental, moral and physical, by virtue of the invigorating influence of an intermingling of race and blood, the favorable hygienic possibilities of her life, and the freedom she is given. But the average girl is not what she might be, and I repeat that this is due to our habits of life and our methods of labor and education.

"EDUCATION."

We must of necessity inquire whether the health of the girl does suffer during school life, or rather, is functional life impaired by our present methods of education, and I must unhesitatingly say that it is unfortunately a question which is asked in different ways and distorted by the partisans of higher education for women.

Statistics of functional health during school life are out of the question, and we must refer to a few general facts, and to our individual observation.

How commonly do we see the once healthy girl returning from school, above all the pernicious boarding school, neurasthenic, with flushed face, cold feet, impaired digestion, backache, painful and disordered functions, all of which symptoms gradually fade with the enjoyment and recreation of vacation, to return with the next session of school.

It is the American idea of putting one through, which lays the foundation of evils which not only follow the individual through life, but pursue her in her descendants. It is the idea of finishing her education, a given job to be completed before the pleasures of society can be anticipated, almost invariably fatal for the bright gifted girl in her last year at school. It is the source of beginning functional disturbances. She is straining every nerve for the dangerous struggle of supremacy, and, cost what it may, her ambition must be gratified ; she must graduate with honor before making her *debut* in society, and even now her emotional nature is stimulated and excited by the foretaste of its pleasures. Evenings which should be devoted to rest are given to the boy visitors and dancing parties. Healthy recreations, outdoor exercises and the necessary sleep are neglected ; school gymnastics, calisthenics or official recreation do not afford the healthful exercises needed. An increased quantity of blood it diverted to the brain, whilst the general supply is diminished and the circulation impaired ; lassitude, malaise and local trouble follow. Is it to be wondered at that she breaks down ? that the mothers best fitted to produce capable children fail to fulfil their destiny ?

In boarding schools enervating routine takes the place of social dissipation, but the results are the same, if not worse, as the girl is removed from her natural guardian and adviser—the mother. One statement from the pen of Dr. Goodel will best indicate the frequency of the injury. He tells us that he has been repeatedly asked by physicians attending such institutions whether it were possible that laundresses could have drugged the scholars unknown to them, in order to avoid the washing of soiled napkins ? So common is the complete cessation of that essential function in the most critical period in the girl's life, removed from home influence with a view of securing the supposed better advantages.

This is the state of affairs in common schools and bo ing schools, at the most critical epoch, from twelve to eighteen; now let us see what the results of so-called higher education are: the showing is a better one as we may see from the report of the Association of College Alumni who have investigated the present health of female college alumnæ as far as practicable.

The health of these girls was very much the same as that of their parents, but three per cent. better, constitutional weakness being mostly the cause of such disorders as did exist. Overwork, accidents and bad sanitary conditions would explain others. Among the college graduates the deterioration in health is two and one-half per cent. less than in the working girls of Boston at the same time.

Worry over studies alone causes no decline in health, whilst worry over personal affairs caused decline of health in ten per cent., and worry over studies and personal affairs combined, in fifteen per cent., but the health of those who declined in health in college has more than recovered in later years.

We find but a very slight falling off, and that only from excellent to fair health in college life, as we might expect if we consider that it is only the healthier and stronger girls who venture upon higher education, and that one-half of these avoided exertion during the menstrual period and saw but little society.

We must bear in mind that college education is an innovation of recent date, in the establishment of which modern hygiene has been consulted, and study and recreation, as well as the health of the pupil, are subject to constant medical supervision; moreover, in some of the more advanced institutions good health is made a condition of admission, and yet Miss Howe, of the college alumni committee, finds that only four hundred and ninety-six out of one thousand graduates married between the ages of fifteen and sixty, and she comes to the conclusion that the tendency of higher education for women is to celibacy; if this is by choice or necessity—for reasons moral or physical—she does not say; it appears to be the natural result of misdirected culture.

The injurious effects of our present female education upon the essential function of woman must be apparent if we bear in mind the period of life from twelve to eighteen, and eighteen to twenty-one, when nutrition should be directed to the essential organs of female life, whilst all other organs are in active growth, likewise demanding increased supply; it is then that an increased expenditure of vital energy is demanded, and the brain concentrates upon itself the nutrient fluid; it is at this time when the system is most susceptible to disturbing influences of all kinds and in an almost explosive state during one week each month, that it is subjected to the greatest strain, to over brain work, nervous and emotional excitement and even physical injuries.

Are the results not natural when we consider that girls in this dangerous period of life spend more time in study than boys, and that boys do have healthy recreation, that the Greeks even withheld male children from study until the tenth year, while laying a solid foundation for a healthy physical system and a harmonious development of the functions.

INJURIES ARE WROUGHT IN OUR SYSTEMS OF EDUCATION AND LABOR.

Very similar are the injurious effects of our systems of labor upon the developing girl and the reproductive functions, but less occult and more marked than those of education, for the reason that the unfortunate sufferer cannot withdraw like the school girl at will, or when the evidences of injury are distinctly felt. She is obliged to continue until she is prostrated.

It is not manual labor only, it is not alone wear and tear on muscle which tells; nerve wear is still worse. It is the girl in the employments now so much affected, as a so-called higher class of female labor, in telephone and telegraph offices; the clerk, the type-setter and the stenographer. Examples of this kind we unfortunately see every day.

A most interesting report is given by the Bureau of Labor statistics of 1875, on the special effect of certain forms of employment on female health, from which I shall quote, as it is the first, perhaps, which has regarded the cardinal relation which labor bears to essential attributes of the forming women, on which hinge all other vital results.

The alarm bell, the first evidence of coming trouble is menstrual disturbance, and how rapidly nerve strain reacts upon the functions, we may see by the example of unexpected rush of business suddenly befalling an operator, in good health, during the period of functional activity, the complete cessation of the flow, the general prostration which followed, with slow and imperfect recovery.

In the main these functional disturbances are produced by overwork, with innutritious and non-sanitary associations, and labor of both body and mind, and the effect of disease primarily produced by early employ during animal growth, the regular and long continued employ of the plastic, undeveloped girl, and the long day's work with unremitting attention. As causative errors in the management of labor are mentioned in this most admirable report the following:

1. A: Youth unequal to the work.
   B: Impairment of animal growth.
   C: A constrained position.
2. A: A disregard of ultimate injuries.
   B: Unbroken application without vacation for long term.
   C: Depression and disease inviting demand on immature vitality.
3. Employ in unsuitable occupation for condition of body and mind.
4. A: Unduly long hours.
   B: Concentration of vital energies, involving extreme nerve tension.
   C: Unfavorable sanitary surrounding.

KNOWLEDGE OF FUNCTION.

Constant injury is wrought by the error of system in school-room and work-shop, but potent and more directly evident causes of ill health and functional disturbance in the growing girl exist in our daily life, our social customs, and our habits of dress. To them I will not refer; they are too well known.

The constriction and compression of the corset, the dragging pounds of the skirt, the circulation impeding garter, the insufficiencies of low necked dresses, of thin stockings and shoes, and the total absence of protection where it is most needed, the absence of drawers.

Among our social customs there are many which bring her ruin. I cannot even touch upon them all; there is but one of which I shall speak, and that is the most dangerous of all—more or less underlying all other causes of disease; it is the ignoring of the function of woman by woman, by the mother, and her ignorance of its import.

Fearful are the consequences of woman's ignorance; the calamities which follow the course of the misguided mother, swift, certain and lasting; the penalties inflicted upon the unadvised or ill-advised girl, whose one great misfortune is ignorance. She steps into the unknown sphere of womanhood, and in darkness she pursues its irregular path; fortunate she who may by chance not stumble.

Many who might be saved by proper management during the transition from adolescence to maturity, now fall victims to their ignorance, and no words of mine could be more convincing than the data given by Tilt as far back as 1853, in his admirable work on the elements of health and the principle of female hygiene, which shows how many are injured by the unexpected appearance of the unknown function.

Great is the danger in all classes, be it from ignorance or modesty, and most susceptible is the more highly strung nervous system of the more refined organization. Even though the bark float in safety through that first stormy epoch of life, it is constantly endangered, most of all from the ceaseless crash of the ever occurring waves of functional activity, and it remains in need of guidance until it has passed through a final storm into calmer waters. The mother is the pilot, and functional hygiene the guiding chart; the physician the engineer who maps it out.

I cannot exaggerate the danger to the delicate bark, to the health of the susceptible girl, from each wave of functional activity. Its greater danger is during the height of the wave. That period of greatest activity throughout the entire system, the period of vascular pressure and nerve excitement, which threaten her functions, as well as the era marked by local depletion and depression, the decline of the wave.

So common are the injuries to female health at this epoch that I need but state a few of the examples which have come under my observation during the writing of this paper, in fact I may say almost upon this very day, to astound you. Fright, nervous and emotional excitement, exposure to cold have brought injury to so many at this time. What more natural than that the anxious girl should seek to check the bleeding wound, as she supposes?

For this purpose the use of cold washes and applications is common; some even seek to stop the flow by a cold bath, as was done by a now careful mother, who lay long at the point of death from the result of such indiscretion, and but slowly by care, regained her health. The terrible warning has not been lost, and, mindful of her own experience, she has taught her children a lesson which but few are fortunate enough to learn.

How many who have passed in safety through the first ordeal are ruined by an indiscretion or an exposure at a later period? Many a vigorous frame has been broken, health has been ruined and death even caused, by disturbances of the function during its period of activity by exposure, by cold, by physical and mental exertion, by nerve or emotional excitement. A cold foot-bath during the period, a dance in a low-neck dress, a walk in rain or snow, a hard day's work of mind or muscle, an excitement of heart or head, has made an invalid of many a previously healthy girl, by this influence on this omnipotent function.

### CONCLUSION.

I have endeavored to show that the health of the American girl is threatened and impaired by causes more or less avoidable, as they are due to our methods of life, our methods of training and education; that the physique of this girl most favorably situated, amid auspicious possibilities, is imperfect, her brain overworked, her nerve power exhausted, her function impaired, and reproduction endangered; all by reason of the susceptibility of her peculiar organization, and the increased impressionability of the sensitive system during the years of development, in which it is subjected to the most severe strain.

Such is the fact. What is the remedy ? Condition and cause make the remedy self-evident.

Let me briefly review the conditions as we have found them : a perfectly organized being, in the very beginning of woman's existence, waning upon the rise of the great wave of functional life, during the period of functional development, in labor and education indicative of nerve and physical prostration ; with impaired circulation and digestion, imperfect menstruation, and diminished reproductive power ; neurasthenia and functional disturbances constantly intermingled as cause and effect ; results brought about, in the main, by more or less the same influences : 1. Over brain work and nerve strain, with neglect of the physical system to education. 2. Nerve strain and partial or incomplete muscular activity in labor ; both influences which are inseparably connected with, and complicated by, causes more active and independently potent. 3. The ignoring and neglecting of functional hygiene. 4. Physical and emotional strain of society, improprieties of dress and over stimulation of the senses.

The remedy is : attention to woman's peculiar organization and the cyclical waves of her dominant function ; or in other words, harmonious development and occupation of nerve and muscle ; diminished brain work and nerve stimulation, with increased and co-ordinate physical exercise ; increased protection and diminished compression of dress ; self-knowledge and individual care during periods of heightened susceptibility.

Changes are necessary in custom and fashion, in methods of labor and education.

Whilst each individual, and each calling, is a law unto itself, I may say, in a general way, that we should endeavor to obtain the end of education in its widest sense, the development of all functions and facilities, to render the girl fit for the life she is to enter, in every way; "to render youth beautiful, healthful, strong and honest."

A harmonious co-education of mind and body should be approximated, with coincident maintenance of proper hygienic conditions. The nerve and emotion strain of class competition must be abolished ; the stress of constant work, the train of thought and the routine of regulation must be broken ; mind and heart should be educated, rather than memory, the nerve strain varied by healthful pleasures and physical exercises in the open air, and relieved more or less, according to individual necessities, during periods of heightened susceptibility.

Whilst the initial causes of ill health in the school girl may readily be overcome, the dangers which beset the laboring girl, though equally evident, are more difficult of removal. The same necessity exists for individual care, upon the height of the functional wave and during its period of decline ; the same necessity for a proper co-operation of labor, physical and mental ; the same danger from constant application, from the strain of one part, one function or organ to the exclusion of others ; nerve tension is even more continuous and intense, and muscular exertion limited to individual muscles.

The year of development should be respected, and the continuity of labor broken, rest and change afforded frequently, for short periods. Much good might be done by the necessary changes in customs and fashions, by suitable dress, but I will not venture an attack upon so powerful a force, which will in time gradually yield to the good sense of the American girl.

I will close with a plea for the self-care of the girl and her proper physiological instruction by the mother, which, alone, will mitigate or remove the initial cause of many of her ailments. Upon the mother I wish to impress that the perfect development of the female function, and the maintenance of this function, once developed, in healthy condition, is essential to the perfect development of the girl and the perfect health of the woman ; that self-care, a well regulated female hygiene, is the foundation of her well-being, and that it is the mother's first duty to so guard herself and so guard her daughter.

